# The role of sea ice for vascular plant dispersal in the Arctic

**DOI:** 10.1098/rsbl.2016.0264

**Published:** 2016-09

**Authors:** Inger Greve Alsos, Dorothee Ehrich, Marit-Solveig Seidenkrantz, Ole Bennike, Andreas Joachim Kirchhefer, Aslaug Geirsdottir

**Affiliations:** 1 UiT—The Arctic University of Tromsø, Tromsø, Norway; 2 Centre for Past Climate Studies, and Arctic Research Centre, Department of Geoscience, Aarhus University, Aarhus, Denmark; 3 GEUS, Copenhagen, Denmark; 4 Dendroøkologen, Skogåsvegen 6, NO-9011 Tromsø, Norway; 5 Faculty of Earth Sciences, University of Iceland, Reykjavík, Iceland

**Keywords:** Arctic, climate change, plant colonization, plant dispersal, driftwood, sea ice

## Abstract

Sea ice has been suggested to be an important factor for dispersal of vascular plants in the Arctic. To assess its role for postglacial colonization in the North Atlantic region, we compiled data on the first Late Glacial to Holocene occurrence of vascular plant species in East Greenland, Iceland, the Faroe Islands and Svalbard. For each record, we reconstructed likely past dispersal events using data on species distributions and genetics. We compared these data to sea-ice reconstructions to evaluate the potential role of sea ice in these past colonization events and finally evaluated these results using a compilation of driftwood records as an independent source of evidence that sea ice can disperse biological material. Our results show that sea ice was, in general, more prevalent along the most likely dispersal routes at times of assumed first colonization than along other possible routes. Also, driftwood is frequently dispersed in regions that have sea ice today. Thus, sea ice may act as an important dispersal agent. Melting sea ice may hamper future dispersal of Arctic plants and thereby cause more genetic differentiation. It may also limit the northwards expansion of competing boreal species, and hence favour the persistence of Arctic species.

## 1. Introduction

The ongoing climate change is expected to cause a northward expansion of the range of various species of plants and animals [1–3]. Oceans form significant barriers for the spread of terrestrial species, yet, in the Arctic, sea ice may act as a ferry, transporting biological material such as terrestrial debris, driftwood, and sometimes propagules attached to driftwood [4,5]. It may also act as a bridge allowing mammals to cross between land areas, potentially carrying viable propagules in their intestine or fur [5–7]. Moreover, smaller organisms and seeds may be blown by wind across sea ice [5,8–11]. However, the currently decreasing sea-ice cover [12] may reduce this natural dispersal agent and thereby limit connectivity in the Arctic and prevent future range shift. We therefore need an improved understanding of the importance of sea ice as a dispersal agent to better forecast the future of Arctic populations and communities needed for adequate management and conservation decisions.

Some of the most remote Arctic islands are found in the North Atlantic sector, which has also been identified as one of the major barriers to dispersal of Arctic-alpine plants [13,14]. Despite this ocean barrier, species distributions and phylogenetic data indicate that the frequency of dispersal events in this region is higher than farther south [8]. The only dispersal agent specific to the Arctic is sea ice. Moreover, dispersal routes often follow the routes of ocean surface currents and sea-ice movement [8,9]. Thus, it has been hypothesized that sea ice has played a central role in Holocene colonization of islands in the North Atlantic region [8,9]. Whereas the northern North Atlantic sea-ice extent was generally larger than today throughout the Late Glacial (15 000–11 700 cal yr BP) and early Holocene (11 700–8000 cal yr BP, [15–17]) when much of the plant colonization took place, there have been no attempts to directly connect sea-ice data at the time of colonization to plant dispersal.

Here, we compile data on the first Late Glacial to Holocene occurrence of vascular plant species, likely dispersal routes, as well as reconstructions of past sea-ice extent to assess the importance of sea ice in facilitating plant dispersal during postglacial re-colonization. Driftwood has limited floating capacity in open water but may be transported over long distances by sea ice [4]. Thus, it represents a visible indicator of long-range ice-driven dispersal, and we compiled data on driftwood as an independent indication for the potential role of sea-ice for plant colonization.

## 2. Methods

### (a) Compilation of data

We compiled data on first occurrences of plant taxa on Svalbard, Iceland, East Greenland and the Faroe Islands based on macrofossils, pollen and/or ancient DNA that were identified to species level or could be ascribed to such because only one species within the genera is currently present in the region (electronic supplementary material, table S1).

The past sea-ice pathways were estimated based on a compilation of published sea-ice proxy records (e.g. [18]) from the study region (electronic supplementary material), quantified through a sea-ice index ranging from 0 (absent) to 9 (perennial). It should be noted that these categories are only general estimates and not precise data. Ocean currents and wind directions were taken into account when estimating sea-ice pathways (electronic supplementary material, table S2).

For driftwood, we compiled published records from the Amphi-Atlantic region dated and traced by means of dendrochronology (electronic supplementary material).

### (b) Statistical analysis

We used the first occurrences of plant species to create two datasets. For a subset of species (*genetic dataset*), colonization patterns in the North Atlantic had previously been inferred on the basis of phylogeographic analysis of amplified fragment length polymorphism [8]. In that study, individuals from colonized areas (target) were assigned to potential source regions with an assignment test. In this study, we used the number of individuals assigned to each possible source area (two to five observations per first occurrence) as response variable in a general linearized mixed model with a Poisson distribution. Sea-ice indices and the log of distance between source and target were explanatory variables, and the identity of each observation was included as random effect (electronic supplementary material).

All retrieved first occurrences were included in the *floristic dataset*. For each record, all potential colonization routes in the North Atlantic were listed and classified as ‘potential’ if the species occurred in the source region, otherwise ‘not likely’. A ‘most likely’ colonization route was determined in each case either based on genetic data (see above) or based on the dominant colonization direction for the target area inferred by Alsos *et al.* [8] (electronic supplementary material). We tested for a correlation of sea ice and most common colonization routes by assessing the difference in average sea-ice index between routes that were classified as ‘not likely’, ‘potential’ or ‘most likely’.

## 3. Results

First occurrence records were found for 36, 52, 31 and 18 plant species from East Greenland, Iceland, Svalbard and the Faroe Islands, respectively. The corresponding median of the first occurrences were 9550, 11 050, 8700 and 11 000 cal yr BP. At that time, sea ice commonly connected most regions along the inferred dispersal routes (electronic supplementary material, table S2).

**Figure 1.**
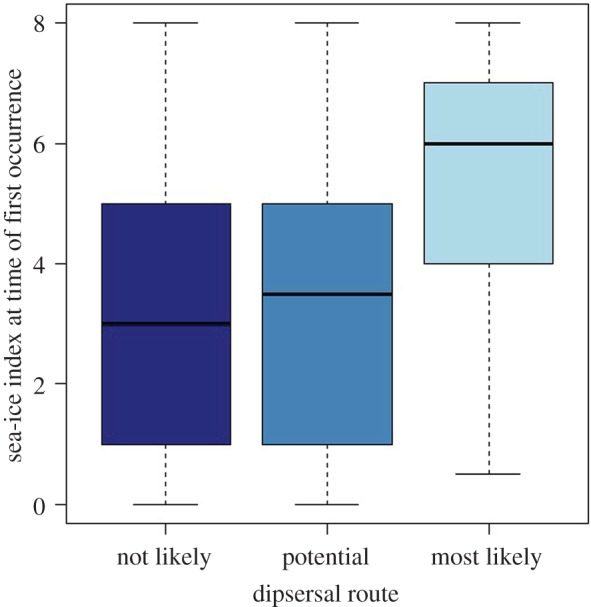
Boxplot of sea ice densities along ‘not likely’, ‘potential’ and ‘most likely’ plant dispersal routes based on 137 first occurrence data of 102 species, which each have six to eight potential dispersal routes, thus a total of 901 considered pathways. The sea-ice indices range from 0 (absence of sea ice) through 4 (occasional, less than 1 month per year) to 8 (dense sea ice). The middle lines show the median, boxes indicate the interquartile range and whiskers the range of the data. The sea ice was significantly denser along the ‘most likely’ dispersal routes than along routes classified as ‘not likely’ or ‘potential’ (*p* < 0.001).

Based on the analysis of the *genetic dataset* (14 first occurrences from eight species), the number of individuals assigned to the respective source region increased 2.22 times for an increase of one in sea-ice index (95% confidence interval (CI) = 1.03–5.68; *p* = 0.049), whereas distance had a significant negative effect (electronic supplementary material). Moreover, analysis of the *floristic dataset* revealed that sea ice was significantly denser along the ‘most likely’ dispersal routes than along routes classified as ‘not likely’ or ‘potential’ (increase in average sea-ice index 2.53, CI = 2.05–3.02; *p* < 0.001; figure 1).

**Figure 2.**
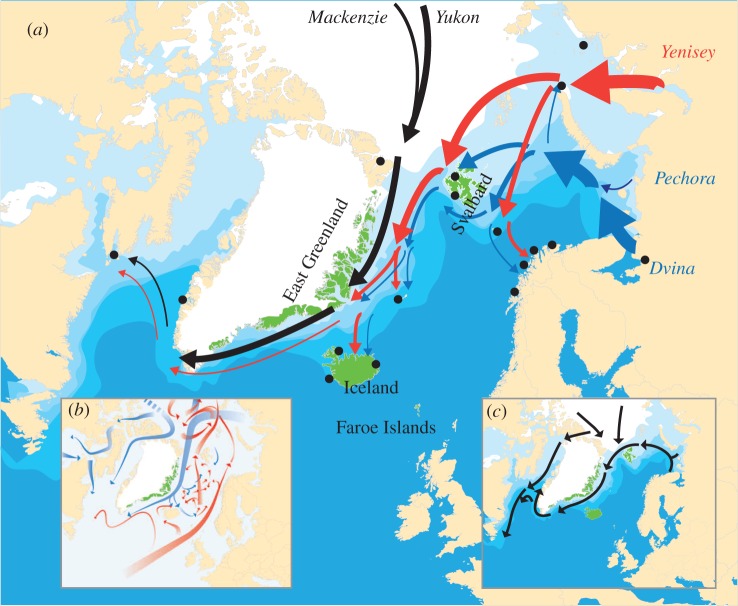
(*a*) The three main dendrochronologically inferred routes of driftwood in the North Atlantic region based on a compilation of sources (electronic supplementary material). Arrow thickness indicates the relative frequency of driftwood dispersal along that route, arrow colours indicate main source areas, and dots indicate sampling sites. Current sea ice from https://nsidc.org/. (*b*) Ocean surface currents from [8]. (*c*) Dominant sea-ice pathways as controlled by dominant wind and ocean currents today. It is, however, notable that short-term variability in sea ice especially linked to shifts in wind patterns and ocean gyres may result in sea-ice transport directions that diverge from the dominant pattern (e.g. [19]).

The compilation of driftwood data (electronic supplementary material) shows that the presence of driftwood mainly is confined to regions with sea ice. Further, the inferred driftwood routes mainly follow the sea-ice and surface ocean current patterns (figure 2).

## 4. Discussion

Overall, the denser sea ice along the most likely colonization routes, and the close link between driftwood and sea-ice distribution, strongly support that sea ice has been and probably still is an important vector for dispersal of plants to Arctic islands.

While the presence of sea ice at the first time of occurrence strongly indicates the importance of this vector, it does not preclude dispersal by other means such as wind, birds and ocean currents [20–22]. The inference based on genetic data, that several source regions contributed to the colonization of the Atlantic Islands, indeed suggests that several different dispersal agents played a role [8]. Generally, mammals and birds are assumed to be less important for dispersal in the Arctic than at lower latitudes owing to their overall lower densities and the low number of passerines [5]. Reindeer may cross sea ice of up to 380 km [6] and geese migrate in huge numbers to the Arctic each year. However, they are not likely to have played a major role for the early plant colonization as they rely on vascular plants as food. Foxes may have arrived earlier, having been able to cross the sea ice to almost all Arctic islands, including Iceland [6,23]. Nevertheless, the traveling time to, e.g. Svalbard may be higher than the retention time, limiting their role as seed dispersers [7]. There were no terrestrial mammals prior to the arrival of humans on the Faroe Islands, and only foxes on Iceland and Jan Mayen, reducing the chance of dispersal by mammals to these islands. Wind, on the other hand, can be strong in the Arctic, and may, especially in combination with sea ice, be a strong dispersal mechanism. Despite the strong link between ocean currents, sea ice and wind, short-term shifts in winds (weather) may also provide the means of dispersal in directions different from the overall prevailing wind patterns.

Both dispersal [10,14] and climatic constraints [9] have been suggested to limit the distribution of vascular plant species in the Arctic today. It is, therefore, likely that the ongoing reduction of sea ice will reduce dispersal in the Arctic and thereby affect community dynamics. Thus, while climate warming may favour growing conditions for many more warmth-requiring species [9], melting sea ice may limit their chance to get there. Consequently, inhibition of the northwards migration of northern boreal species may limit the role of the Arctic as a climate refugium for them. This lack of competition from boreal species may in fact favour the persistence of the Arctic flora in a warming climate. However, lower connectivity may lead to higher genetic differentiation and eventually speciation as seen by different subspecies of the Arctic fox in the southernmost islands [23]. Also, as re-colonization may be limited, stochastic extinction may increase especially on small islands.

## Supplementary Material

Late Glacial to Holocene additional data

## Data accessibility

The datasets and details of data assembling supporting this article have been uploaded as the electronic supplementary material.
