# Supplementary material for: The role of sea ice for vascular plant dispersal in the Arctic
Source: Biol Lett. 2016 Sep;12(9):20160264. doi: 10.1098/rsbl.2016.0264 (PMC5046916; doi:10.1098/rsbl.2016.0264)
Supplement: Late Glacial to Holocene additional data [file rsbl20160264supp1.docx]

# Supplementary material to:

# The role of sea ice for vascular plant dispersal in the Arctic

Inger Greve Alsos, Dorothee Ehrich, Marit-Solveig Seidenkrantz, Ole Bennike, Andreas Joachim Kirchhefer, Aslaug Geirsdottir

*Compilation of first occurrences data of vascular plants*

We reviewed all published sources of Late Glacial to Holocene records of vascular plant occurrences that we were aware of for Svalbard, Iceland, East Greenland, the Faroe Islands, and Jan Mayen and recorded first occurrences of species in each of these target regions. Jan Mayen was subsequently excluded from the analyses, as we found no published records from there. We also excluded first occurrences from records that had major hiatus or lacked data from the early ice-free period of that region, or where the chronologies were not up to modern standards. We used calibrated published dates where available or otherwise calibrated the recorded ^14^C dates using Calib 7.1 (<http://calib.qub.ac.uk/calib/calib.html>) with IntCal13 [[1](#_ENREF_1)]. We follow the most updated taxonomy for the region [[2](#_ENREF_2)]. All first records used are given in Table S1.

*Estimates of sea ice density and pathway*

Modern sea ice extent and thickness are estimated from satellite images, providing detailed information on regional and seasonal differences (National Snow and Ice Data Center, USA, <https://nsidc.org/>). However, satellite-based sea ice cover estimates only reach back to 1979, and for the pre-instrumental times, estimates of sea ice cover and transport paths mainly rely on information from the geological record.

Geological records that provide information on past sea-ice cover result primarily from sea-ice proxies in marine sediment records (sediment cores retrieved from the sea floor), but also driftwood, beach ridges, as well as to some extent lake sediment cores and ice cores, may provide some information. Proxies that indicate sea-ice cover include biological, geochemical and sedimentological parameters, each with different strengths and weaknesses/uncertainties. For a detailed description of these proxies, see [[3](#_ENREF_3)].

In the present study, the sea-ice information through time was based on information from a wide range of previously published marine sediment records from the study region (e.g. [[3-7](#_ENREF_3)]), dividing the region into possible pathways for sea-ice transport (e.g. from East Greenland to Iceland, from Svalbard to East Greenland etc.) (Table S2). For the last 10,000 cal. years BP, sea-ice data was compiled from average information representing 2000-year time slices, whereas the period 15,000-10,000 years was divided into 1000-yr time slices due to the significant change in climate during this time period. The sea-ice cover for each pathway and each time slice was classified into categories, each representing a general estimate: 0) absence of sea ice, 2) rare (almost never), 4) occasional (< 1 month/year), 6) common (winter, 1-6 month/year), 8) dense (6-10 month/year), and 9) perennial (11-12 month/year) according to groups defined by Seidenkrantz et al. ( [[8](#_ENREF_8)]; unpublished). In addition, as each region/pathway was often only represented by a few sites making the sea-ice distribution data less precise, intermediate categories were also used: 1) no to very rare, 3) rare/occasional, 5) occasional/common (winter), and 7) common/dense. The sea-ice index at time of first occurrence of species for potential pathways (Table S1) are given in Table S2. It should, however, be kept in mind that these data are rough estimates and that the sea ice categories are not based on precise data.

Today transport of sea ice is primarily determined by surface ocean circulation and wind patterns and strengths. Thus, for each of the studied time slices, the sea-ice cover and its potential as a medium for transport of plant material over longer distances was evaluated based on both the extent and concentration of sea ice as well as the potential transport paths allowed by ocean currents and atmospheric circulation. Hence, when taking ocean current into account, the main Holocene sea-ice transport pathways would be northwards from northern Norway to Spitsbergen and southwards along the coast of Greenland and towards Iceland. The warm currents of the North Atlantic Drift system (Figure 2 ) today act as a barrier for sea ice and would likely also have somewhat limited the sea-ice transport between Iceland, Faroe Islands and Britain/Scandinavia as well as between Spitsbergen and Greenland in the early Holocene. However, the expanded sea-ice cover during the Younger Dryas may have facilitated an increased sea-ice transport also between these sites. Wind-driven transport of plant material over sea ice is less limited to specific directions due to the more chaotic pattern of the atmospheric circulation, although the transport direction will still be dominated by primary wind directions, i.e. the westerlies at mid latitudes and the polar easterlies at higher latitudes.

*Dispersal pathways based on genetic and floristic data*

Data sets were assembled by assuming that all areas surrounding the north Atlantic and where the species is growing today could be the source for colonization of the target areas (Table S1). Dispersal routes over land (for instance from West or North Greenland to East Greenland) were not considered as we were interested in relating the importance of potential dispersal pathways to sea ice. For species where the past dispersal routes had been estimated based on genetic data (Amplified fragment length polymorphism; *genetic dataset),* the importance of each source region was inferred from an assignment test carried out in AFLPOP [11] and the source to which most individuals had been assigned was considered the maximum likely source region (see Figure 1 in [[2](#_ENREF_2)]). If genetic groups extended over several regions (e.g. Great Britain, Norway and northern Russia, the sea-ice data were used from the northernmost regions (thus in general the highest likelihood of sea ice). This regards especially colonisation of Iceland and the Faroe Islands, where the genetic data in most cases did not allow distinction between Great Britain, Norway or both as source regions. Moreover, areas were only included as potential source regions after the first occurrence of plants in that region. Thus, Svalbard, the Faroe Islands and East Greenland were only included as potential sources regions for dispersal since 10,000, 11,200 and 11,800 cal. years BP, respectively.

For the species which had not been analysed genetically (*floristic dataset*), we assumed that the plants had followed the main colonization route to that region, determined on the basis of genetic data in previous studies. Main source regions for each target region are given in Table S2. If a species was not found in the main source region today, it was assumed to have dispersed from the second most likely source region.

For East Greenland, we could only test for the role of sea ice for dispersal from the east as the dispersal route to the west is largely hampered by the Greenland Ice Sheet [[2](#_ENREF_2)]. The proportion of western species is higher in North and South Greenland but decreases towards the central parts of East Greenland [[9-11](#_ENREF_9)], indicating that dispersal from West to central East Greenland may have been dominated by a gradual migration over land rather than by long-distance dispersal. Therefore, for species from East Greenland, which were absent from Russia, we did not specify a most likely source region.

For Iceland and the Faroe Islands, it was most often not possible to distinguish between Great Britain, South or North Scandinavia as the main source region [[2](#_ENREF_2)], and we therefore performed alternative analyses (see below). Because of their geographic situation, East Canada and South Scandinavia were not considered among the direct sources for colonization of Svalbard. The first case would indeed most probably imply colonization over land over Greenland and the second case over North Scandinavia. East Canada was also not considered a source for the Faroe Islands.

We here only estimated sea­-ice density at first record on islands, despite the fact that the true colonisation is more complex due to e.g. back-dispersal, recurrent dispersal and extinction. Based on Holocene records of species that no longer exist on the islands today, e.g. *Betula nana* on the Faroe Island [[12](#_ENREF_12)] and *Angelica* cf. *archangelica* and *Dryas* on Bjørnøya, Svalbard [[13](#_ENREF_13)], we know that there is a certain extinction rate. Detailed studies from the newly formed island of Surtsey 9 km south of Iceland show that the extinction rate may be very high especially in the early colonization process [[14](#_ENREF_14)], and it is likely to be higher for small islands. The re-colonisation rate is likely to increase with island size and decrease with dispersal distance [[15](#_ENREF_15)]. Thus, if re-colonisation happened frequently, our use of sea-ice index at age of first record is likely to overestimate the average density of sea ice at time of colonization. However, as most species probably arrived prior to their first record in palaeo-studies, we are more likely to have underestimated the sea ice density.

*Statistical analysis including supplementary results*

The *genetic dataset* comprised 40 dispersal routes relating to 14 first occurrence dates for 8 species in total (*Angelica archangelica, Betula nana, Betula pubescens, Dryas octopetala, Empetrum nigrum, Juniperus communis, Micranthes stellaris, Salix herbacea*), and included all four target regions. The data were analysed using a generalized linear mixed effects model (GLMM) with the number of individuals assigned to a particular source region by the assignment test as response variable. The assignment tests had previously been carried out using AFLPOP [[16](#_ENREF_16)]. We used a Poisson distribution, the number of individuals analysed as offset, the natural logarithm (log) of the distance between source and target and the sea ice index as fixed factors, and the specific colonization event as random effect on the intercept. The residuals of this model showed overdispersion. We resolved this problem by including the individual observation as additional random effect.

The floristic dataset comprised in total 901 possible dispersal routes for 102 species and 137 first occurrence dates. For Iceland and the Faroe Islands, the most likely source region determined in genetic studies was Scandinavia/Great Britain [[2](#_ENREF_2)]. As sea-ice conditions differed considerably between North Scandinavia and South Scandinavia/Great Britain, statistical analyses were carried out for both scenarios, taking North Scandinavia and South Scandinavia as the most likely source (Figures 1 and S1, respectively). The differences in sea ice score between the three groups of routes (“most likely”, “potential” and “not likely” was assessed with a linear model using these groups as explanatory factor. The source region classified as “most likely” had on average a higher sea-ice index than the other sources, also when assuming that colonization from Scandinavia occurred from South Scandinavia (Figure S1). The sea ice index along dispersal routes classified as “most likely” was in this case on average 1.58 higher than for “not likely” (95% CI = 1.08 – 2.09; p < 0.001). “Potential” routes had slightly higher sea ice indices than “not likely” routes, but the difference was not significant: 0.07 (CI= -0.27 – 0.42; p = 0.68) for northern Scandinavia and 0.32 (CI= -0.04 – 0.68; p = 0.08) for southern Scandinavia as main sources.

*Estimating relative importance of driftwood routes*

The natural source of Arctic driftwood today are the boreal forests along the rivers draining into the Arctic Ocean, including the Barents and Bering Seas. The major source areas are the vast, forested catchments of the Siberian rivers. Trees and shrubs on riverbanks, together with sediments and biological material potentially including seeds, can be uprooted and transported downstream by river ice and flood events. Already Kjellman (1882) and Ingmarson (1903) found seeds in crevasses of driftwood on Novaya Zemlya and East-Greenland, respectively, thus proving that driftwood can be a transport medium for propagules [[17](#_ENREF_17), [18](#_ENREF_18)]. Subarctic and boreal woods stay afloat up to six (birch and willows) and 17 months (spruce) before getting water-logged and sinking [[19](#_ENREF_19)]. This is not sufficient for travelling long distances and in order to cross the Arctic Ocean, logs must be captured by sea ice. For final deposition, logs must be released in open water and washed ashore. The last part of its journey thus can be affected by surface ocean currents and strong winds. As an example, the arrival of larger quantities of driftwood in northern Norway is often related to northerly storms.

Natural logs can easily be identified by the presence of roots. Today, and probably during the past 200 years, these are outnumbered by timber that is lost during floating, i.e., timber transport on rivers. During the 19^th^ century, lost cargo from vessels also played a certain role in the Barents and Norwegian Sea. Despite of this fact, the present driftwood is an important indicator of transport mechanisms and routes. The transport routes have changed over time {Funder, 2011 #3586}{Dyke, 1997 #3702}. Especially, the opening of the Bering Land Bridge may have change the currents. However, that occurred 11 000 year ago so mainly before the colonization studied here. Both the Beaufort Gyre and the Transpolar drift are assumed to have existed through the period studied here, so the changes in currents for the amphi-Atlantic region have probably mainly been in the relative contribution rather than complete change of transport direction.

Part of recent driftwood has been dendrochronologically dated. By this means, also their origin is revealed. For instance, logs on Baffin Island have been traced back to the Yukon and Mackenzie, and logs on Iceland to the Angara region in Central Siberia close to Mongolia [[20](#_ENREF_20)]. However, the origin of many driftwood samples has not been tracked, yet, possibly because the trees were short-lived or due to the lack of reference chronologies for certain regions and time periods. In these cases, the wood species still can indicate a potential growth region. Based on the species distribution and river discharges, one can assume that, e.g., most of recent, still undated larch logs on Arctic beaches derive from Siberia (Hellmann et al. 2015).

For this study, we compiled only records on dendrochronologically dated and provenanced driftwood [[20-27](#_ENREF_20)]. Driftwood that has been subject of wood-identification only, were not included because their assignment to origin is less specific (see e.g, [[28](#_ENREF_28)]).

An indicator that driftwood and sea ice played an important role also in the early Holocene, is the presence of 9000 year old driftwood in northern Greenland [[29](#_ENREF_29)] and 11,000 year old driftwood on Spitsbergen and Franz-Joseph’s Land [[30](#_ENREF_30), [31](#_ENREF_31)]), respectively. Modern analogues to potential early Holocene sea-ice and/or driftwood transport between the source region of the British Isles and Norway and the target region of the Faroe Islands and Iceland are lacking. However, considering differences in the shore lines, river drainage, sea ice distribution, sea surface currents and possibly wind directions might allow the conclusion that sea-ice and driftwood also were important factors in this sector of the study region. In Germany and Switzerland, pine logs recovered from river gravels were dendrochronologically dated back to 10,461-7942 BC and 12,410-9891 BP, respectively [[32](#_ENREF_32)]. This shows that, e.g., the rivers Rhine and Elbe could supply tree stems that under suitable circumstances could be transported to Iceland – if not straight across the North Atlantic Current, probably by means of sea-ice moving counter-clockwise around a partially ice-free Norwegian Sea and approaching Iceland from the north-east.

## Figure legend

Figure S1. Boxplot of sea ice densities along “not likely”, “potential”, and “most likely” plant dispersal routes based on 137 first occurrence data of 102 species, which each have 6-8 potential dispersal routes, thus a total of 901 considered pathways. The sea-ice indices range from 0 (absence of sea ice), through 4 (occasional, <1 month per year) to 8 (dense sea ice). The middle lines show the median, boxes indicate the interquartile range and whiskers the range of the data. South Scandinavia is assumed as most likely source for Iceland and the Faroe Islands (see text).

## References

[1] Reimer, P.J., Bard, E., Bayliss, A., Beck, J.W., Blackwell, P.G., Bronk Ramsey, C., Buck, C.E., Cheng, H., Edwards, R.L., Friedrich, M., et al. 2013 *IntCal13 and marine13 radiocarbon age calibration curves 0–50,000 years cal BP*.

[2] Alsos, I.G., Ehrich, D., Eidesen, P.B., Solstad, H., Westergaard, K.B., Schönswetter, P., Tribsch, A., Birkeland, S., Elven, R. & Brochmann, C. 2015 Long-distance plant dispersal to North Atlantic islands: colonization routes and founder effect. *AoB Plants* **7**. (doi:10.1093/aobpla/plv036).

[3] de Vernal, A., Gersonde, R., Goosse, H., Seidenkrantz, M.-S. & Wolff, E.W. 2013 Sea ice in the paleoclimate system: the challenge of reconstructing sea ice from proxies – an introduction. *Quat. Sci. Rev.* **79**, 1-8. (doi:<http://dx.doi.org/10.1016/j.quascirev.2013.08.009>).

[4] Massé, G., Rowland, S.J., Sicre, M.-A., Jacob, J., Jansen, E. & Belt, S.T. 2008 Abrupt climate changes for Iceland during the last millennium: Evidence from high resolution sea ice reconstructions. *Earth Planet. Sci. Lett.* **269**, 565-569. (doi:<http://dx.doi.org/10.1016/j.epsl.2008.03.017>).

[5] Pearce, C., Seidenkrantz, M.-S., Kuijpers, A. & Reynisson, N.F. 2014 A multi-proxy reconstruction of oceanographic conditions around the Younger Dryas–Holocene transition in Placentia Bay, Newfoundland. *Mar. Micropaleontol.* **112**, 39-49. (doi:<http://dx.doi.org/10.1016/j.marmicro.2014.08.004>).

[6] Müller, J. & Stein, R. 2014 High-resolution record of late glacial and deglacial sea ice changes in Fram Strait corroborates ice–ocean interactions during abrupt climate shifts. *Earth Planet. Sci. Lett.* **403**, 446-455. (doi:<http://dx.doi.org/10.1016/j.epsl.2014.07.016>).

[7] Sha, L., Jiang, H., Seidenkrantz, M.-S., Muscheler, R., Zhang, X., Knudsen, M.F., Olsen, J., Knudsen, K.L. & Zhang, W. 2016 Solar forcing as an important trigger for West Greenland sea-ice variability over the last millennium. *Quat. Sci. Rev.* **131, Part A**, 148-156. (doi:<http://dx.doi.org/10.1016/j.quascirev.2015.11.002>).

[8] Seidenkrantz, M.-S., de Vernal, A., Goosse, H., S., S., Van Nieuwenhove, N., Macias-Fauria, M., Klein, F., Pearce, C., Belt, S., Caissie, B., et al. 2014 Arctic sea-ice cover during the Holocene – proxy data reconstruction and modelling. In *American Geophysical Union, Fall Meeting* (San Fransico.

[9] Bay, C. 1992 A phytogeographical study of vascular plants of northern Greenland - north of 74º northern latitude. *Medd. Grønl. Biosci.* **36**, 1-102.

[10] Feilberg, J. 1984 A phytogeographical study of South Greenland. Vascular plants. *Medd. Grønl. Biosci.* **15**, 1-72.

[11] Böcher, J., Holmen, K. & Jakobsen, K. 1959 A synoptical study of the Greenland flora. *Medd. Grønl.* **163**, 1-32.

[12] Johansen, J. 1975 Pollen diagrams from the Shetland and Faroe Islands. *New Phytol.* **75**, 369-387.

[13] Wohlfarth, B., Lemdahl, G., Olsson, S., Persson, T., Snowball, I., Ising, J. & Jones, V. 1995 Early Holocene environment on Bjørnøya (Svalbard) inferred from multidisciplinary lake sediments studies. *Polar Res.* **14**, 253-275.

[14] Magnússon, B., Magnússon, S.H., Ólafsson, E. & Sigurdsson, B.D. 2014 Plant colonization, succession and ecosystem development on Surtsey with reference to neighbouring islands. *Biogeosciences* **11**, 5521-5537. (doi:10.5194/bg-11-5521-2014).

[15] Weigelt, P. & Kreft, H. 2013 Quantifying island isolation – insights from global patterns of insular plant species richness. *Ecography* **36**, 417-429. (doi:10.1111/j.1600-0587.2012.07669.x).

[16] Duchesne, P. & Bernatchez, L. 2002 AFLPOP: a computer program for simulated and real population allocation, based on AFLP data. *Mol Ecol. Notes* **2**, 380-383. (doi:10.1046/j.1471-8278 .2002.00251.x).

[17] Kjellman, F.R. 1882 Fanerogamfloraen på Novaja Semlja och Wajgatsch. Växtgeografisk studie. In *Vega-expeditionens vetenskapliga iakktagelser. Bearbetade af deltagare i resan och andra forskara* (ed. Nordenskiöld), pp. 321-352. Stockholm, Beijers Förlag.

[18] Ingvarson, F. 1903 Om drifveden i Norra Ishafvet. *Kungl. Sv. Vetensk.-Akad. Handl.* **37**, 1-84.

[19] Häggblom, A. 1982 Driftwood in Svalbard as an indicator of sea ice conditions. *Geogr. Ann. A* **64**, 81-94.

[20] Johansen, S. 1998 The origin and age of driftwood on Jan Mayen. *Polar Res.* **17**, 125-146. (doi:10.1111/j.1751-8369.1998.tb00267.x).

[21] Bartholin, T.S. & Hjort, C. 1987 Dendrochronological studies of recent driftwood on Svalbard. In *Task force meeting on methodology of dendrochronology, Methods of dendrochronology* (eds. L. Kairiukstis, Z. Bednarz & E. Feliksik), pp. 207-220 Krakow, International Institute for Applied Systems Analysis| Polish Academy of Sciences; Systems Research Institute.

[22] Eggertsson, Ó. 1993 Origin of the driftwood on the coasts of Iceland: A dendrochronological study. *Jökull* **43**, 15-32.

[23] Eggertsson, Ó. 1994 Driftwood as an indicator of relative changes in the influx of Arctic and Atlantic water into the coastal areas of Svalbard. *Polar Res.* **13**, 10. (doi:10.3402/polar.v13i2.6694).

[24] Eggertsson, Ó. 1994 Mackenzie River Driftwood: A Dendrochronological Study. *Arctic* **47**, 128-136. (doi:10.14430/arctic1282).

[25] Eggertsson, Ó. & Laeyendecker, D. 1995 A dendrochronological study of the origin of driftwood in Frobisher Bay, Baffin Island, N.W.T., Canada. *Arct. Alp. Res.* **27**, 180-186.

[26] Johansen, S. 1999 Origin of driftwood in north Norway and its relevance for transport routes of drift ice and pollution to the Barents Sea. *Sci. Total Environ.* **231**, 201-225.

[27] Johansen, S. 2001 A dendrochronological analysis of driftwood in the Northern Dvina delta and on northern Novaya Zemlya. *J. Geophys. Res.* **106**, 19929-19938. (doi:10.1029/1999jc000023).

[28] Hellmann, L., Tegel, W., Kirdyanov, A.V., Eggertsson, Ó., Esper, J., Agafonov, L., Nikolaev, A.N., Knorre, A.A., Myglan, V.S., Churakova, O., et al. 2015 Timber logging in Central Siberia is the main source for recent arctic driftwood. *Arct. Antarct. Alp. Res.* **47**, 449-460. (doi:doi:10.1657/AAAR0014-063).

[29] Funder, S., Goosse, H., Jepsen, H., Kaas, E., Kjær, K.H., Korsgaard, N.J., Larsen, N.K., Linderson, H., Lyså, A., Möller, P., et al. 2011 A 10,000-year record of Arctic Ocean sea-ice variability—View from the beach. *Science* **333**, 747-750.

[30] Salvigsen, O. 1984 Occurrence of pumice on raised beaches and Holocene shoreline displacement in the inner Isfjorden area, Svalbard. *Polar Res.* **2**, 107-113. (doi:10.3402/polar.v2i1.6964).

[31] Weihe, R. 1996 Late Quaternary glacial geology and relative sea level history of Franz Josef Land, Russia. The Ohio State University.

[32] Friedrich, M., Remmele, S., Kromer, B., Hofmann, J., Spurk, M., Kauser, K.F., Orcel, C. & Kuppers, M. 2004 The 12,460-year Hohenheim oak and pine tree-ring chronology from Central Europe; a unique annual record for radiocarbon calibration and paleoenvironment reconstructions. *Radiocarbon* **46**, 1111-1122.

[33] Rundgren, M. 1998 Early-Holocene vegetation of northern Iceland: pollen and plant macrofossil evidence from the Skagi peninsula. *Holocene* **8**, 553-564.

[34] Wagner, B., Bennike, O.L.E., Cremer, H. & Klug, M. 2010 Late Quaternary history of the Kap Mackenzie area, northeast Greenland. *Boreas* **39**, 492-504. (doi:10.1111/j.1502-3885.2010.00148.x).

[35] Bennike, O., Björck, S., Böcher, J., Hansen, L., Heinemeier, J. & Wohlfarth, B. 1999 Early holocene plant and animal remains from North-east Greenland. *J. Biogeogr.* **26**, 667-677.

[36] Birks, H.H. 1991 Holocene vegetational history and climatic changes in west Spitsbergen - plant macrofossils from Skardtjørna, an Arctic lake. *The Holocene* **1**, 209-218.

[37] Rundgren, M. & Ingólfsson, O. 1999 Plant survival in Iceland during periods of glaciation? *J. Biogeogr.* **26**, 387-396.

[38] Fredskild, B. 1995 Palynology and sediment slumping in a high arctic Greenland lake. *Boreas* **24**, 345-354.

[39] Funder, S. 1978 Holocene stratigraphy and vegetation history in the Scoresby Sund area, East Greenland. *Bulletin Grønlands Geologiske Undersøgelse* **129**, 1-66.

[40] Alsos, I.G., Sjögren, P., Edwards, M.E., Landvik, J.Y., Gielly, L., Forwick, M., Coissac, E., Brown, A.G., Jakobsen, L.V., Føreid, M.K., et al. 2016 Sedimentary ancient DNA from Lake Skartjørna, Svalbard: Assessing the resilience of arctic flora to Holocene climate change. *The Holocene* **26**, 627-642. (doi:10.1177/0959683615612563).

[41] Hallsdóttir, M. & Caseldine, C. 2005 The Holocene vegetation history of Iceland, state-of-the-art and future research. In *Modern Processes and Past Environments* (eds. C. Caseldine, A. Russell, J. Hardardóttir & O. Knudsen). Amsterdam, Elsvier.

[42] Caseldine, C., Langdon, P. & Holmes, N. 2006 Early Holocene climate variability and the timing and extent of the Holocene thermal maximum (HTM) in northern Iceland. *Quat. Sci. Rev.* **25**, 2314-2331. (doi:<http://dx.doi.org/10.1016/j.quascirev.2006.02.003>).

[43] Caseldine, C., Geirsdóttir, À. & Langdon, P. 2003 Efstadalsvatn - a multi-proxy study of a Holocene lacustrine sequence from NW Iceland. *J. Paleolimnol.* **30**, 55-73.

[44] Bennike, O.L.E. & Weidick, A. 2001 Late Quaternary history around Nioghalvfjerdsfjorden and Jøkelbugten, North-East Greenland. *Boreas* **30**, 205-227. (doi:10.1111/j.1502-3885.2001.tb01223.x).

[45] Bennike, O. & Hedenäs, L. 1995 Early Holocene land floras and faunas from Edgeøya, Eastern Svalbard. *Polar Res.* **14**, 205-214.

[46] Wagner, B. & Bennike, O. 2015 Holocene environmental change in the Skallingen area, eastern North Greenland, based on a lacustrine record. *Boreas* **44**, 45-59. (doi:10.1111/bor.12085).

[47] Hannon, G.E., Rundgren, M. & Jessen, C.A. 2010 Dynamic early Holocene vegetation development on the Faroe Islands inferred from high-resolution plant macrofossil and pollen data. *Quaternary Res.* **73**, 163-172. (doi:<http://dx.doi.org/10.1016/j.yqres.2009.11.003>).

[48] Bennike, O. & Wagner, B. 2012 Deglaciation chronology, sea-level changes and environmental changes from Holocene lake sediments of Germania Havn Sø, Sabine Ø, northeast Greenland. *Quaternary Res.* **78**, 103-109. (doi:<http://dx.doi.org/10.1016/j.yqres.2012.03.004>).

[49] Hallsdóttir, M. 1987 Pollen analytical studies of human influence on vegetation in relatin to the landnám tephra layer in Southwest Iceland [Lundqua Thesis 18].

[50] Bennike, O. 2010 Palaeoecology of Skálafjørdur, the Faroe Islands. *Ann. Soc. Sci. Færoensis, suppl.* **52**.

[51] Bennike, O. 1999 Colonisation of Greenland by plants and animals after the last ice age: a review. *Polar Rec.* **35**, 323-336.

## Supplementary tables

**Table S1**. First occurrence of vascular plant species in Greenland, Iceland, Svalbard and the Faroe Islands based on our compilation of palaeorecords. Also, their present occurrence in the Amphi-Atlantic region is given according to [[2](#_ENREF_2)].

| Taxon | E Greenland | E Greenland first | E Greenland reference | Iceland | Iceland first | Iceland reference | Svalbard | Svalbard first | Svalbard reference | Faroe Islands | Faroe Islands first | Faroe Islands reference | Jan Mayen | N Scandinavia | S Scandinavia | Fennoscandia | E Canada | W Greenland | N Greenland | N Ural Mts. |
| --- | --- | --- | --- | --- | --- | --- | --- | --- | --- | --- | --- | --- | --- | --- | --- | --- | --- | --- | --- | --- |
| *Angelica archangelica* L. subsp. *archangelica* |  |  |  | 1 | 12000 | [[33](#_ENREF_33)] |  |  |  |  |  |  |  | 1 | 1 | 1 |  | 1 |  | 1 |
| *Angelica sylvestris* L. | 1 |  |  | 1 | 12000 | [[33](#_ENREF_33)] |  |  |  | 1 |  |  |  | 1 | 1 | 1 |  |  |  | 1 |
| *Arabis alpina* L. | 1 | 10400 | [[34](#_ENREF_34), [35](#_ENREF_35)] | 1 |  |  | 1 | 9000 | [[36](#_ENREF_36)] | 1 |  |  | 1 | 1 | 1 | 1 | 1 | 1 |  | 1 |
| *Armeria maritima* (Mill.) Willd. |  |  |  | 1 | 12500 | [[37](#_ENREF_37)] |  |  |  | 1 |  |  |  | 1 | 1 | 1 |  | 1 |  |  |
| *Armeria scabra* Pall. ex Roem. & Schult. | 1 | 9800 | [[38](#_ENREF_38)] |  |  |  |  |  |  |  |  |  |  |  | 1 | 1 | 1 | 1 | 1 | 1 |
| *Betula nana* L. subsp. *nana* | 1 | 8800 | [[39](#_ENREF_39)] | 1 | 13000 | [[37](#_ENREF_37)] |  |  |  |  |  |  | 1 | 1 | 1 | 1 |  | 1 |  | 1 |
| *Betula nana* L. var. *tundrarum* (Perfil.) Elven |  |  |  |  |  |  | 1 | 7491 | [[40](#_ENREF_40)] |  |  |  |  |  |  |  |  |  |  | 1 |
| *Betula pubescens* Ehrh. subsp. *tortuosa* (Ledeb.) Nyman |  |  |  | 1 | 12000 | [[33](#_ENREF_33)] |  |  |  |  |  |  |  | 1 | 1 | 1 |  |  |  | 1 |
| *Bistorta vivipara* (L.) Delarbre | 1 | 7900 | [[34](#_ENREF_34), [35](#_ENREF_35)] | 1 | 11000 | [[41](#_ENREF_41)] | 1 | 9000 | [[36](#_ENREF_36)] | 1 |  |  | 1 | 1 | 1 | 1 | 1 | 1 | 1 | 1 |
| *Botrychium lunaria* (L.) Sw. | 1 |  |  | 1 | 11000 | [[42](#_ENREF_42)] | 1 |  |  | 1 |  |  | 1 | 1 | 1 | 1 | 1 | 1 |  | 1 |
| *Braya glabella* Richardson subsp. *purpurascens* (R.Br.) Cody | 1 | 10100 | [[38](#_ENREF_38)] |  |  |  | 1 |  |  |  |  |  |  |  | 1 | 1 | 1 | 1 | 1 | 1 |
| *Calamagrostis neglecta* (Ehrh.) P.Gaertn., B.Mey & Scherb. subsp. *groenlandica* (Schrank) Matuszk. |  |  |  | 1 |  |  | 1 | 7227 | [[40](#_ENREF_40)] |  |  |  |  | 1 | 1 | 1 | 1 | 1 | 1 | 1 |
| *Callitriche palustris* L. |  | 7800 | [[35](#_ENREF_35)] | 1 |  |  |  |  |  |  |  |  |  | 1 | 1 | 1 | 1 | 1 |  | 1 |
| *Calluna vulgaris* (L.) Hull |  |  |  | 1 | 7600 | [[43](#_ENREF_43)] |  |  |  | 1 | 10400 | [[12](#_ENREF_12)] |  | 1 | 1 | 1 |  |  |  |  |
| *Caltha palustris* L. subsp. *radicans* (T.F.Forst.) Hook. | 1 |  |  | 1 | 13000 | [[37](#_ENREF_37)] |  |  |  | 1 | 11000 | [[12](#_ENREF_12)] | 1 | 1 | 1 | 1 | 1 |  |  | 1 |
| *Cardamine bellidifolia* L. |  |  |  | 1 |  |  | 1 | 2931 | [[40](#_ENREF_40)] |  |  |  |  | 1 | 1 | 1 | 1 | 1 | 1 | 1 |
| *Carex bigelowii* Torr. ex Schwein. subsp. *bigelowii* | 1 | 9000 | [[34](#_ENREF_34), [35](#_ENREF_35)] |  |  |  |  |  |  |  |  |  |  | 1 | 1 | 1 | 1 | 1 | 1 |  |
| *Carex concolor* R.Br. (*C.* *aquatilis* Wahlenb. subsp. *stans* (Drejer) Hultén) | 1 | 8800 | [[44](#_ENREF_44)] |  |  |  | 1 |  |  |  |  |  |  | 1 | 1 | 1 | 1 | 1 | 1 | 1 |
| *Carex fuliginosa* Schkuhr subsp. *misandra* (R.Br.) Nyman | 1 | 10200 | [[38](#_ENREF_38)] |  |  |  | 1 |  |  |  |  |  |  | 1 | 1 | 1 | 1 | 1 |  | 1 |
| *Cassiope tetragona* (L.) D.Don | 1 | 7800 | [[34](#_ENREF_34)] |  |  |  | 1 |  |  |  |  |  |  |  | 1 | 1 | 1 | 1 | 1 | 1 |
| *Cerastium arcticum* Lange | 1 | 9700 | [[38](#_ENREF_38)] |  |  |  | 1 | 10000 | [[45](#_ENREF_45)] |  |  |  |  |  |  |  | 1 | 1 | 1 |  |
| *Chamerion latifolium* (L.) Holub |  | 10000 | [[38](#_ENREF_38)] | 1 |  |  |  |  |  |  |  |  |  |  |  |  | 1 | 1 | 1 | 1 |
| *Cochlearia groenlandica* L. |  |  |  | 1 |  |  | 1 | 8325 | [[40](#_ENREF_40)] |  |  |  |  |  |  |  | 1 | 1 | 1 | 1 |
| *Cryptogramma crispa* (L.) R.Br. ex Hook. | 1 |  |  | 1 | 10300 | [[43](#_ENREF_43)] |  |  |  |  |  |  |  | 1 | 1 | 1 |  |  |  | 1 |
| *Diphasiastrum alpinum* (L.) Holub | 1 |  |  | 1 | 11500 | [[43](#_ENREF_43)] |  |  |  | 1 | 11000 | [[12](#_ENREF_12)] | 1 | 1 | 1 | 1 | 1 | 1 |  | 1 |
| *Dryas integrifolia* Vahl | 1 | 6700 | [[46](#_ENREF_46)] |  |  |  |  |  |  |  |  |  |  |  |  |  | 1 | 1 | 1 |  |
| *Dryas octopetala* L. | 1 | 7800 | [[34](#_ENREF_34), [35](#_ENREF_35)] | 1 | 13000 | [[37](#_ENREF_37)] | 1 | 10000 | [[45](#_ENREF_45)] | 1 |  |  | 1 | 1 | 1 | 1 |  |  |  | 1 |
| *Eleocharis palustris* (L.) Roem. & Schult. s.l. | 1? |  |  | 1 |  |  |  |  |  | 1 | 10400 | [[12](#_ENREF_12)] |  | 1 | 1 | 1 | 1 | 1 |  |  |
| *Empetrum nigrum* L. 2x |  |  |  | 1 | 13000 | [[37](#_ENREF_37)] |  |  |  | 1 | 11200 | [[47](#_ENREF_47)] |  | 1 | 1 | 1 | 1 |  |  |  |
| *Empetrum nigrum* L. 4x (*E*. *hermaphroditum* Hagerup p.p.) | 1 | 10400 | [[35](#_ENREF_35)] | 1 | 13000 | [[37](#_ENREF_37)] | 1 |  |  | 1 |  |  | 1 | 1 | 1 | 1 | 1 | 1 | 1 | 1 |
| *Equisetum variegatum* Schleich. ex F.Weber & D.Mohr | 1 | 7900 | [[35](#_ENREF_35)] | 1 |  |  | 1 |  |  | 1 |  |  | 1 | 1 | 1 | 1 | 1 | 1 | 1 | 1 |
| *Eriophorum scheuchzeri* Hoppe subsp. *arcticum* M.S.Novos. | 1 | 9900 | [[48](#_ENREF_48)] |  |  |  | 1 |  |  |  |  |  |  |  |  |  | 1 | 1 | 1 | 1 |
| *Filipendula ulmaria* (L.) Maxim. |  |  |  | 1 | 11000 | [[42](#_ENREF_42)] |  |  |  | 1 | 11000 | [[12](#_ENREF_12)] |  | 1 | 1 | 1 |  |  |  |  |
| *Geranium sylvaticum* L. | 1 |  |  | 1 | 8000 | [[49](#_ENREF_49)] |  |  |  | 1 |  |  |  | 1 | 1 | 1 |  | 1 |  | 1 |
| *Gymnocarpium dryopteris* (L.) Newman | 1 |  |  | 1 | 9000 | [[49](#_ENREF_49)] |  |  |  | 1 |  |  |  | 1 | 1 | 1 | 1 | 1 |  | 1 |
| *Harrimanella hypnoides* (L.) Coville |  | 7800 | [[35](#_ENREF_35)] | 1 |  |  | 1 | 8000 | [[36](#_ENREF_36)] |  |  |  |  | 1 | 1 | 1 | 1 | 1 |  | 1 |
| *Hippuris vulgaris* L. | 1 | 9400 | [[35](#_ENREF_35)] | 1 | 10600 | [[43](#_ENREF_43)] |  |  |  |  |  |  |  | 1 | 1 | 1 | 1 |  |  | 1 |
| *Huperzia arctica* (Grossh. ex Tolm.) Sipliv. |  |  |  | 1 |  |  | 1 | 8460 | [[40](#_ENREF_40)] |  |  |  |  | 1 | 1 | 1 | 1 | 1 | 1 | 1 |
| *Huperzia selago* (L.) Bernh. ex Schrank & Mart. | 1 |  |  | 1? | 11200 | [[43](#_ENREF_43)] |  |  |  |  |  |  |  | 1 | 1 | 1 | 1 | 1 |  | 1 |
| *Isoëtes echinospora* Durieu |  |  |  | 1 | 9500 | [[42](#_ENREF_42)] |  |  |  | 1 | 11000 | [[12](#_ENREF_12)] |  | 1 | 1 | 1 |  |  |  |  |
| *Isoëtes lacustris* L. |  |  |  | 1 | 8500 | [[49](#_ENREF_49)] |  |  |  | 1 | 11000 | [[12](#_ENREF_12)] |  | 1 | 1 | 1 |  | 1 |  |  |
| *Juncus biglumis* L. |  |  |  | 1 |  |  | 1 | 5131 | [[40](#_ENREF_40)] | 1 |  |  |  | 1 | 1 | 1 | 1 | 1 | 1 | 1 |
| *Juniperus communis* L. subsp. *communis* | 1 |  |  | 1 | 13000 | [[37](#_ENREF_37)] |  |  |  |  |  |  |  | 1 | 1 | 1 |  |  |  | 1 |
| *Juniperus communis* L. subsp. *nana* Syme |  |  |  | 1 | 13000 | [[37](#_ENREF_37)] |  |  |  | 1 | 11000 | [[12](#_ENREF_12)] |  | 1 | 1 | 1 |  | 1 |  | 1 |
| *Koenigia islandica* L. |  |  |  | 1 | 12500 | [[37](#_ENREF_37)] | 1 | 8325 | [[40](#_ENREF_40)] | 1 |  |  |  | 1 | 1 | 1 | 1 | 1 | 1 | 1 |
| *Linum catharticum* L. |  |  |  | 1 | 11200 | [[43](#_ENREF_43)] |  |  |  | 1 |  |  |  | 1 | 1 | 1 |  |  |  |  |
| *Littorella uniflora* (L.) Asch. |  |  |  | 1 | 9000 | [[49](#_ENREF_49)] |  |  |  | 1 |  |  |  | 1 | 1 | 1 |  |  |  |  |
| *Lycopodium annotinum* L. subsp. *annotinum* | 1 |  |  | 1 | 12300 | [[37](#_ENREF_37)] |  |  |  |  |  |  | 1 | 1 | 1 | 1 | 1 | 1 |  | 1 |
| *Lycopodium clavatum* L. subsp. *clavatum* | 1 |  |  | 1 | 11500 | [[43](#_ENREF_43)] |  |  |  |  |  |  |  | 1 | 1 | 1 | 1 |  |  | 1 |
| *Menyanthes trifoliata* L. subsp. *trifoliata* | 1 |  |  | 1 | 10000 | [[41](#_ENREF_41)] |  |  |  | 1 | 10200 | [[12](#_ENREF_12)] |  | 1 | 1 | 1 |  |  |  | 1 |
| *Micranthes stellaris* (L.) Galasso, Banfi & Soldano |  |  |  | 1 | 11250 | [[43](#_ENREF_43)] |  |  |  | 1 |  |  |  | 1 | 1 | 1 | 1 | 1 |  |  |
| *Minuartia rubella* (Wahlenb.) Hiern |  | 10200 | [[38](#_ENREF_38)] | 1 |  |  | 1 | 10000 | [[45](#_ENREF_45)] | 1 |  |  |  | 1 | 1 | 1 | 1 | 1 | 1 | 1 |
| *Montia fontana* L. |  |  |  | 1 | 10800 | [[43](#_ENREF_43)] |  |  |  | 1 |  |  |  | 1 | 1 | 1 | 1 | 1 |  |  |
| *Myriophyllum alterniflorum* DC. | 1 |  |  | 1 | 11100 | [[43](#_ENREF_43)] |  |  |  | 1 | 11000 | [[12](#_ENREF_12)] |  | 1 | 1 | 1 | 1 | 1 |  |  |
| *Myriophyllum sibiricum* Kom. |  |  |  | 1 | 11300 | [[43](#_ENREF_43)] |  |  |  |  |  |  |  | 1 | 1 | 1 | 1 | 1 |  | 1 |
| *Oxyria digyna* (L.) Hill | 1 |  |  | 1 | 13000 | [[37](#_ENREF_37)] | 1 | 10000 | [[45](#_ENREF_45)] | 1 |  |  | 1 | 1 | 1 | 1 | 1 | 1 | 1 | 1 |
| *Papaver dahlianum* Nordh. | 1 | 11800 | [[35](#_ENREF_35)] |  |  |  | 1 | 10000 | [[45](#_ENREF_45)] |  |  |  |  |  | 1 | 1 | 1 | 1 | 1 | 1 |
| *Papaver radicatum* Rottb. |  |  |  | 1 | 10300 | [[43](#_ENREF_43)] |  |  |  | 1 |  |  |  | 1 | 1 | 1 |  |  |  |  |
| *Parnassia palustris* L. subsp. *palustris* | 1 |  |  | 1 | 10200 | [[41](#_ENREF_41)] |  |  |  |  |  |  |  | 1 | 1 | 1 |  |  |  | 1 |
| *Pedicularis hirsuta* L. | 1 | 7800 | [[35](#_ENREF_35)] |  |  |  | 1 |  |  |  |  |  |  |  | 1 | 1 | 1 | 1 | 1 | 1 |
| *Phippsia concinna* (Th.Fr.) Lindeb. | 1 | 9200 | [[38](#_ENREF_38)] |  |  |  | 1 |  |  |  |  |  |  | 1 |  | 1 | 1 | 1 | 1 | 1 |
| *Pinguicula vulgaris* L. | 1 |  |  | 1 | 9000 | [[41](#_ENREF_41)] |  |  |  | 1 |  |  |  | 1 | 1 | 1 | 1 | 1 |  | 1 |
| *Plantago lanceolata* L. |  |  |  | 1 | 8500 | [[43](#_ENREF_43)] |  |  |  | 1 |  |  |  | 1 | 1 | 1 |  |  |  |  |
| *Plantago maritima* L. | 1 |  |  | 1 | 12000 | [[37](#_ENREF_37)] |  |  |  | 1 | 10500 | [[12](#_ENREF_12)] |  | 1 | 1 | 1 | 1 | 1 |  | 1 |
| *Poa alpina* L. var. *vivipara* L. |  |  |  | 1 |  |  | 1 | 8460 | [[40](#_ENREF_40)] |  |  |  |  | 1 | 1 | 1 |  |  |  | 1? |
| *Polygonum boreale* Lange |  |  |  | 1 | 11000 | [[42](#_ENREF_42)] |  |  |  | 1? |  |  |  |  | 1 | 1 | 1 | 1? |  |  |
| *Populus tremula* L. | 1 |  |  | 1 | 10800 | [[43](#_ENREF_43)] |  |  |  |  |  |  |  | 1 | 1 | 1 |  |  |  | 1 |
| *Potamogeton natans* L. | 1 |  |  | 1 | 10500 | [[43](#_ENREF_43)] |  |  |  | 1 |  |  |  | 1 | 1 | 1 | 1 | 1? |  | 1 |
| *Puccinellia angustata* (R.Br.) E.L.Rand & Redfield | 1 | 9800 | [[38](#_ENREF_38)] |  |  |  | 1 |  |  |  |  |  |  |  |  |  | 1 | 1 | 1 |  |
| *Ranunculus hyperboreus* Rottb. subsp. *arnellii* Scheutz | 1 | 10300 | [[48](#_ENREF_48)] |  |  |  | 1 |  |  |  |  |  |  |  |  |  |  |  |  | 1 |
| *Ranunculus pygmaeus* L. | 1 |  |  | 1 |  |  | 1 | 7700 | [[36](#_ENREF_36)] |  |  |  |  | 1 | 1 | 1 | 1 | 1 | 1 | 1 |
| *Ranunculus sulphureus* Sol. | 1 | 10000 | [[48](#_ENREF_48)] |  |  |  | 1 | 9500 | [[45](#_ENREF_45)] |  |  |  |  |  | 1 | 1 | 1 | 1 | 1 | 1 |
| *Rumex acetosa* L. subsp. *islandicus* (Á.Löve) Ö.Nilsson |  |  |  | 1 |  |  |  |  |  | 1 | 10400 | [[50](#_ENREF_50)] |  |  |  |  |  |  |  |  |
| *Rumex acetosella* L. subsp. *acetosella* | 1 | 7900 | [[35](#_ENREF_35)] | 1 | 13000 | [[37](#_ENREF_37)] |  |  |  |  |  |  |  | 1 | 1 | 1 |  |  |  |  |
| *Rumex acetosella* L. subsp. *arenicola* Y.Mäkinen ex Elven |  |  |  | 1 | 13000 | [[37](#_ENREF_37)] |  |  |  |  |  |  |  | 1 | 1 | 1 | 1? | 1 |  | 1? |
| *Rumex acetosella L. subsp.* *tenuifolius* (Wallr.) O.Schwarz | 1 |  |  | 1 | 13000 | [[37](#_ENREF_37)] |  |  |  |  |  |  | 1 | 1 | 1 | 1 |  |  |  | 1? |
| *Sagina nivalis* (Lindblom) Fr. |  |  |  | 1 |  |  | 1 | 8500 | [[36](#_ENREF_36)] | 1 |  |  |  | 1 | 1 | 1 | 1 | 1 | 1 | 1 |
| *Salix arctica* Pallas |  | 8800 | [[44](#_ENREF_44)] | 1 |  |  |  |  |  | 1 |  |  |  | 1 |  | 1 | 1 | 1 | 1 | 1 |
| *Salix herbacea* L. | 1 | 10800 | [[51](#_ENREF_51)] | 1 | 10900 | [[33](#_ENREF_33)] | 1 | 8000 | [[13](#_ENREF_13)] | 1 | 10400 | [[50](#_ENREF_50)] | 1 | 1 | 1 | 1 | 1 | 1 | 1 | 1 |
| *Salix lanata* L. |  |  |  | 1 |  |  | 1 | 9000 | [[36](#_ENREF_36)] | 1 |  |  |  | 1 | 1 | 1 |  |  |  | 1 |
| *Salix phylicifolia* L. | 1 |  |  | 1 | 9400 | [[33](#_ENREF_33)] |  |  |  | 1 |  |  |  | 1 | 1 | 1 |  |  |  | 1 |
| *Salix polaris* Wahlenb. |  |  |  |  |  |  | 1 | 10000 | [[45](#_ENREF_45)] |  |  |  |  | 1 | 1 | 1 |  |  |  | 1 |
| *Salix reticulata* L. |  |  |  |  |  |  | 1 | 9000 | [[36](#_ENREF_36)] |  |  |  |  | 1 | 1 | 1 | 1 |  |  | 1 |
| *Saxifraga aizoides* L. | 1 |  |  | 1 |  |  | 1 | 9000 | [[36](#_ENREF_36)] |  |  |  | 1 | 1 | 1 | 1 | 1 | 1 | 1 | 1 |
| *Saxifraga cernua* L. | 1 |  |  | 1 |  |  | 1 | 8700 | [[36](#_ENREF_36)] |  |  |  | 1 | 1 | 1 | 1 | 1 | 1 | 1 | 1 |
| *Saxifraga cespitosa* L. |  | 11000 | [[48](#_ENREF_48)] | 1 |  |  | 1 | 8503 | [[40](#_ENREF_40)] |  |  |  |  | 1 | 1 | 1 | 1 | 1 | 1 | 1 |
| *Saxifraga hirculus* L. subsp. *compacta* Hedberg |  |  |  | 1 |  |  | 1 | 5131 | [[40](#_ENREF_40)] |  |  |  |  |  |  |  |  |  |  | 1 |
| *Saxifraga oppositifolia* L. | 1 | 11000 | [[35](#_ENREF_35)] | 1 |  |  | 1 | 10000 | [[45](#_ENREF_45)] | 1 |  |  | 1 | 1 | 1 | 1 | 1 | 1 | 1 | 1 |
| *Saxifraga rivularis* L. subsp. *rivularis* |  |  |  | 1 |  |  | 1 | 9000 | [[36](#_ENREF_36)] | 1 |  |  |  | 1 | 1 | 1 | 1 | 1 | 1 | 1 |
| *Sedum villosum* L. | 1 |  |  | 1 |  |  |  |  |  | 1 | 11000 | [[12](#_ENREF_12)] |  | 1 | 1 | 1 |  | 1 |  |  |
| *Selaginella selaginoides* (L.) P.Beauv. ex Mart. & Schrank | 1 |  |  | 1 | 12100 | [[37](#_ENREF_37)] |  |  |  | 1 | 10400 | [[50](#_ENREF_50)] |  | 1 | 1 | 1 | 1 | 1 |  | 1 |
| *Sibbaldia procumbens* L. | 1 | 8700 | [[35](#_ENREF_35)] | 1 |  |  | 1 |  |  | 1 |  |  | 1 | 1 | 1 | 1 | 1 | 1 |  | 1 |
| *Silene acaulis* (L.) Jacq. | 1 |  |  | 1 |  |  | 1 | 10000 | [[45](#_ENREF_45)] | 1 |  |  | 1 | 1 | 1 | 1 | 1 | 1 | 1 | 1 |
| *Silene sorensenis* (B.Boivin) Bocquet | 1 | 10200 | [[38](#_ENREF_38)] |  |  |  |  |  |  |  |  |  |  |  |  |  | 1 | 1 | 1 |  |
| *Silene uralensis* (Rupr.) Bocquet subsp. *arctica* (Th.Fr.) Bocquet | 1 | 11800 | [[35](#_ENREF_35)] |  |  |  | 1 | 8600 | [[45](#_ENREF_45)] |  |  |  |  |  |  |  | 1 | 1 | 1 | 1 |
| *Sorbus aucuparia* L. | 1 |  |  | 1 | 7000 | [[41](#_ENREF_41)] |  |  |  |  |  |  |  | 1 | 1 | 1 |  |  |  | 1 |
| *Stuckenia filiformis* (Pers.) Börner subsp. *filiformis* |  |  |  | 1 |  |  |  |  |  | 1 | 8000 | [[50](#_ENREF_50)] |  | 1 | 1 | 1 |  |  |  |  |
| *Taraxacum* sect. Arctica | 1 | 7800 | [[35](#_ENREF_35)] |  |  |  | 1 |  |  |  |  |  |  |  |  |  | 1 | 1 | 1 |  |
| *Thalictrum alpinum* L. | 1 |  |  | 1 | 13000 | [[37](#_ENREF_37)] |  |  |  | 1 | 11000 | [[12](#_ENREF_12)] |  | 1 | 1 | 1 | 1 | 1 |  | 1 |
| *Tofieldia pusilla* (Michx.) Pers. | 1 |  |  | 1 | 10500 | [[41](#_ENREF_41)] | 1 |  |  | 1 |  |  | 1 | 1 | 1 | 1 | 1 | 1 |  | 1 |
| *Utricularia minor* L. | 1 |  |  | 1 | 10700 | [[43](#_ENREF_43)] |  |  |  |  |  |  |  | 1 | 1 | 1 | 1 | 1 |  |  |
| *Vaccinium uliginosum* L. | 1 | 7800 | [[35](#_ENREF_35)] | 1 |  |  | 1 |  |  | 1 |  |  | 1 | 1 | 1 | 1 | 1 | 1 | 1 | 1 |

**Table S2**. Sea-ice index between the target regions and source regions at times of first occurrence of vascular plant species (see Table S1). 0) absence of sea ice, 1) no to very rare, 2) rare (almost never), 3) rare/occasional, 4) occasional (< 1 month/year), 5) occasional/common (winter), 6) common (winter, 1-6 month/year), 7) common/dense, 8) dense (6-10 month/year), 9) perennial (11-12 month/year). For each pathway, periods with identical sea-ice conditions are lumped. Dates mentioned for potential source regions indicate the estimated time of deglaciation. These regions were excluded as potential sources for colonization events pre-dating this deglaciation date. After that, for each species, source regions where the species occurs today were classified “potential”, whereas sources regions from where the species is absent today were classified as “unlikely” for the statistical analysis. The source regions regarded as “most likely” based on genetic studies are marked green, whereas regions not included as source regions are marked grey. All dates are given as calibrated years BP.

|  | Target regions | | | |
| --- | --- | --- | --- | --- |
| Source regions | East Greenland | Iceland | Svalbard | Faroe Islands |
| East Greenland  from 11.8 ky |  | 12-10 ky: 5  10-8 ky: 5  8-7 ky: 4 | 11-8 ky: 6  8-2 ky: 5 | 12-11 ky: 6  11-10 ky: 2  10-8 ky: 1  8-7 ky: 0 |
| Iceland  from 13 ky | 12-8 ky: 5  8-6 ky: 4 |  | 11-10 ky: 3  10-6 ky: 4  6-2 ky: 2 | 12-11 ky: 6  11-10 ky: 2  10-8 ky: 1  8-7 ky: 0 |
| Svalbard  from 10 ky | 10-8 ky: 8  8-6 ky: 6 | 10-7 ky: 4 |  | 10-7 ky: 2 |
| Faroes  from 11.2 ky | 11-10 ky: 2  10-8 ky: 1  8-6 ky: 0 | 12-11 ky: 6  11-10 ky: 2  10-8 ky: 1  8-7 ky: 0 | 11-10 ky: 2  10-6 ky: 1  6-2 ky: 0 |  |
| North Scandinavia | 12-8 ky: 6  8-6 ky: 4 | 14-13 ky: 6  13-12 ky: 8  12-11 ky: 6  11-8 ky: 4  8-7 ky: 3 | 11-8 ky: 6  8-2 ky: 4 | 12-11 ky: 6  11-10 ky: 2  10-8 ky: 1  8-7 ky: 0 |
| South Scandinavia | 12-11 ky: 4  11-10 ky: 2  10-8 ky: 1  8-6 ky: 0 | 14-13 ky: 4  13-12 ky: 6  12-11 ky: 4  11-10 ky: 2  10-8 ky: 1  8-7 ky: 0 |  | 12-11 ky: 4  11-10 ky: 2  10-8 ky: 1  8-7 ky: 0 |
| East Canada | 12-10 ky: 7  10-6 ky: 6 | 13-7 ky: 3 |  |  |
| Russia | 12-8 ky: 8  8-6 ky: 6 | 14-13 ky: 6  13-12 ky: 8  12-11 ky: 6  11-8 ky: 4  8-7 ky: 3 | 11-2 ky: 6 | 12-11 ky: 6  11-10 ky: 2  10-8 ky: 1  8-7 ky: 0 |
| Great Britain | 12-6 ky: 0 | 13-7 ky: 0 | 11-2 ky: 0 | 11-2 ky: 0 |

**Table S3.** Parameter estimates with standard error (SE), and p-values for a GLMM with a Poisson distribution modelling the number of plant individuals assigned to a particular source region in function of the log of the distance between target and source (log dist) and the sea-ice index.

| Fixed effect | Estimate | SE | p |
| --- | --- | --- | --- |
| Intercept | 9.02 | 5.92 | 0.401 |
| Log dist | -1.99 | 0.93 | 0.031 |
| Sea ice | 0.80 | 0.41 | 0.049 |

Random effect: individual observation var = 7.88
